# Applied machine learning for predicting the lanthanide-ligand binding affinities

**DOI:** 10.1038/s41598-020-71255-9

**Published:** 2020-08-31

**Authors:** Suryanaman Chaube, Sriram Goverapet Srinivasan, Beena Rai

**Affiliations:** TCS Research, Tata Research Development and Design Center, 54-B Hadapsar Industrial Estate, Hadapsar, Pune, Maharashtra 411013 India

**Keywords:** Chemical engineering, Cheminformatics

## Abstract

Binding affinities of metal–ligand complexes are central to a multitude of applications like drug design, chelation therapy, designing reagents for solvent extraction etc. While state-of-the-art molecular modelling approaches are usually employed to gather structural and chemical insights about the metal complexation with ligands, their computational cost and the limited ability to predict metal–ligand stability constants with reasonable accuracy, renders them impractical to screen large chemical spaces. In this context, leveraging vast amounts of experimental data to learn the metal-binding affinities of ligands becomes a promising alternative. Here, we develop a machine learning framework for predicting binding affinities (*logK*_1_) of lanthanide cations with several structurally diverse molecular ligands. Six supervised machine learning algorithms—Random Forest (RF), k-Nearest Neighbours (KNN), Support Vector Machines (SVM), Kernel Ridge Regression (KRR), Multi Layered Perceptrons (MLP) and Adaptive Boosting (AdaBoost)—were trained on a dataset comprising thousands of experimental values of *logK*_1_ and validated in an external 10-folds cross-validation procedure. This was followed by a thorough feature engineering and feature importance analysis to identify the molecular, metallic and solvent features most relevant to binding affinity prediction, along with an evaluation of performance metrics against the dimensionality of feature space. Having demonstrated the excellent predictive ability of our framework, we utilized the best performing AdaBoost model to predict the *logK*_1_ values of lanthanide cations with nearly 71 million compounds present in the PubChem database. Our methodology opens up an opportunity for significantly accelerating screening and design of ligands for various targeted applications, from vast chemical spaces.

## Introduction

Rare Earth Elements (REEs), that constitute the lanthanide block of the periodic table, together with Yttrium and Scandium, lie at the heart of many modern technologies in diverse fields ranging from health care to clean energy applications^1^. With increasing adoption of clean and energy efficient technologies, the demand for REEs is expected to grow manifold in the coming years^2^. Although conventional mining remains the primary source of global REE supply currently^3^, owing to the huge quantities of electronic waste (e-waste) generated, REE recovery from e-wastes becomes a promising secondary source of these critical elements^4^. Much of the metal processing industry relies upon hydrometallurgical operations such as liquid–liquid extraction (LLE) to recover the target element^5^. The success of an LLE operation depends critically on the choice of ligands that can selectively bind to one or more target metal ions and transport them into an oil phase in contact with an aqueous phase which originally contained the metal ions. Thus, successful recovery of REEs from e-wastes calls for the design of ligands with a high affinity for one or more target lanthanide ions. The binding strength of a ligand to a metal ion depends on a number of factors including the nature of the molecule and the metal ion themselves, the solvent media, ionic strength of the media etc. For ligands that bind via a cation exchange mechanism (such phosphoric acid ligands), pH of the medium further becomes an important factor in determining the binding affinity, since deprotonation of the ligand is a necessary condition for the formation of an M-L complex^6^. Then, any successful design of ligand must necessarily incorporate information about the experimental conditions in addition to the nature of the metal ion itself.

A number of works in the past have attempted to predict the binding affinities of various ligands with different metal ions as well as design ligands that can preferentially bind to one or more target metal ions^7–22^. While molecular modeling using density functional theory could give important chemical insights in addition to binding affinities, the associated computational cost renders this method impractical to screen vast chemical spaces. In such a scenario, leveraging available experimental data on the M-L binding constants to build ‘data-based’ predictive models becomes a promising alternative. Prior works along these lines have predominantly employed Quantitative Structure Property Relations (QSPR) techniques such as Multiple Linear Regression (MLR) to build predictive models for M-L binding constants (Ref.^12^ and the references therein). The ligands were mostly described using the Substructural Molecular Fragments (SMF) descriptors^8,23,24^ and consensus models were developed for each metal ion separately^7–15^. In addition, these works did not consider any properties of the metal ion or the medium while developing the QSPR models. Furthermore, these models were built for either a limited class of ligands or metal ions only. Owing to these restrictions, the errors in these models were relatively high, limiting their generalizability to predict M-L binding constants across vastly different ligand chemistries^7–15^.

In view these limitations, our work employs a machine learning (ML) approach for predicting binding constants of diverse lanthanide-ligand complexes under varied experimental conditions. A total of 698 organic and inorganic ligands were involved in the modelling along with 15 lanthanide cations and 8 solvent media. A host of supervised ML algorithms—Random Forest (RF), k-Nearest Neighbours (KNN), Support Vector Machines (SVM), Kernel Ridge Regression (KRR), Multi Layered Perceptron (MLP) and Adaptive Boosting (AdaBoost)—were trained on an experimental dataset containing 5,266 *logK*_1_ values, validated using a tenfold cross-validation procedure and tested on 1,317 independent *logK*_1_ values. Unlike most previous QSPR studies that have primarily relied on SMF descriptors, the molecular descriptors used in the study comprised both physiochemical (eg. molecular weight) and topological descriptors (eg. topological indices). Also, these descriptors were augmented with properties of metal cation and the solvent medium to factor in experimental conditions, thus enabling better model predictability. A rigorous feature engineering analysis was performed to identify the most relevant features based on three approaches and the findings have been discussed. Subsequently, an out-of-sample model validation was performed on six nitrogen donor ligands with known binding affinity values. Having tested the generalizability of our framework, we employed the best performing regression model to predict binding constants of lanthanide metals with nearly 71 million molecules in the PubChem database, falling within the applicability domain of our models. The demonstrated framework underpins the potential of statistical learning models in accelerating the discovery and development of novel molecular ligands for a target metal extraction from vast chemical spaces.

## Methods

### Dataset generation

The dataset for training our models was generated using the International Union of Pure and Applied Chemistry (IUPAC) Stability Constants Database (SC-database) (provided by Dr. Leslie Pettit). The database contains dissociation/binding/stability constants (in log units) of several metal ions binding with various ligand molecules from reported experimental literature. To begin with, the stability constants (*logK*_1_) for all M-L pairs (M—lanthanide cation, L—ligand, $log{K}_{1}=\frac{\left[ML\right]}{\left[M\right]\left[L\right]}$) were collected and curated from the SC-database. In the subsequent step, we filtered datapoints with available experimental conditions namely temperature, ionic strength and solvent medium, resulting in a total of 6,583 entries. These entries contained 698 unique (ligand) molecules, 15 lanthanide cations—Ce^3+^, Ce^4+^, Pr^3+^, Nd^3+^, Pm^3+^, Sm^3+^, Eu^3+^, Gd^3+^, Tb,^3+^ Dy^3+^, Ho^3+^, Er^3+^, Tm^3+^, Yb^3+^ and Lu^3+^—and 8 solvent media—alcohol, dioxane, KCl, KNO_3_, NaCl, NaClO_4_, NaNO_3_ and R_4_NX. To generate the machine learning descriptors, the corresponding 2D molecular structure files (for all 698 ligands) were downloaded from SC-Database, converted into 3D molecular-data files (‘mol’ format) and fed into RDKit, an open-source cross-platform chemoinformatics toolkit^25^. The tool has a built-in functionality for generating both compositional descriptors like *MolWt*, *NumValenceElectrons*, *NumHDonor* etc. and topological molecular descriptors like *BalabanJ*, *FpDensityMorgan1, PEOE_VSA1* etc. Each 3D molecular structure was optimized using the general purpose UFF force field^26^; the optimized files were then read by the *Chem.Descriptors* module of RDKit to compute nearly 200 available molecular descriptors for each molecule in the database. Out of these, a number of descriptors were removed which were either redundant or null-valued for most of the entries. Some examples include descriptors like *fr_Ar_COO*, *fr_Ar_OH*, *fr_COO2* etc. which only count the number of functional groups of a certain type, an information captured in other compositional descriptors like *MolWt*, *HeavyAtomMolWt*, *NumValenceElectrons* etc. as well. Post this screening, 83 molecular descriptors remained, which were augmented with 14 readily available properties of metal atoms (atomic number, outer shell electrons, ionization energies I–III, electron affinity, atomic radius, covalent radius, ionic radius, Pauling electronegativity, melting point, boiling point, density and standard entropy), 3 properties of solvent medium (density, molar mass and melting point) and experimental conditions, namely temperature and ionic concentration. Octanol and methylammonium chloride were used to represent the alcohol and R_4_NX media, respectively. Like other works in the past^7–12,14–16^, no descriptors based on the structural features of the metal–ligand (M-L) complex (such as denticity, coordination geometry etc.) were included since models built using such features will be inapplicable to predict the logK_1_ values in cases (such as predicting binding affinities across vast chemical spaces) where the M-L structure is unavailable. Thus, the final dataset for training had 102 descriptors for 6,583 datapoints containing known experimental log K_1_ values. The same has been uploaded in the supporting information.

For preprocessing, we implemented six scaling techniques inbuilt in the *scikit-learn* machine learning library of Python^27^. Subsequently, a host of ML models, namely RF, KNN, SVM, KRR, MLP and AdaBoost were trained on the dataset in view of the recent successes of neural networks and kernel-based methods in accelerated material property predictions^28–33^. The details on preprocessing and model training are provided in the supporting information.

## Results

**Figure 1:**
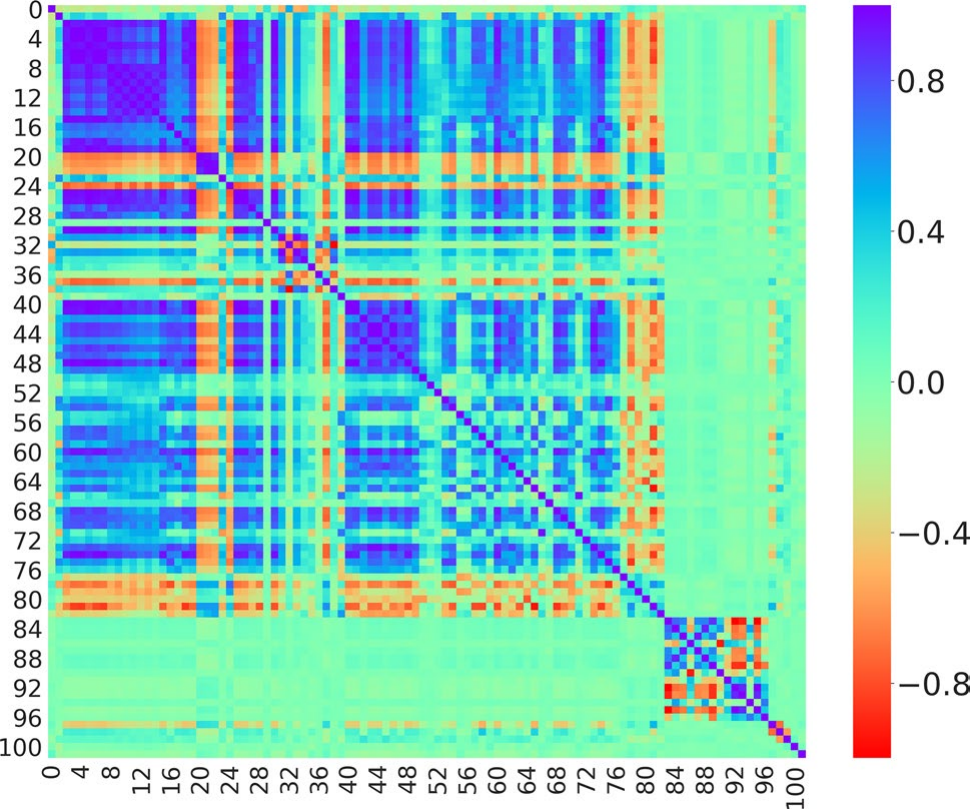
Pearson correlation map depicting the correlation between the features.

Figure 1 depicts the Pearson correlation coefficient matrix representing all 102 features plotted using the Seaborn library of Python^34^. The features, largely speaking, are not highly correlated with understandable exceptions like a few subtypes of the same molecular descriptors, e.g. *Chi* (features 3–14), *Lipinski* parameters (features 40–50) etc. and certain correlated metal properties, e.g. atomic number, outer shell electrons, ionization energies (features 82–90) etc.

### Evaluation of different models and feature engineering

Table 1 lists the optimized model parameters, the corresponding error metrics—Mean Absolute Error (MAE), Root Mean Square Error (RMSE) and the coefficient of determination (R^2^)—and the normalization method employed. It could be inferred that the AdaBoost model demonstrated an exceptional performance on the test dataset (MAE = 0.39, RMSE = 0.91, R^2^ = 0.98) followed closely by the KRR (Laplacian) and RF models (MAE = 0.43 and MAE = 0.44, respectively). Interestingly, either (a) augmenting the features by incorporation of fragment descriptors that were initially eliminated (like *fr_Ar_COO*, *fr_Ar_OH*, *fr_COO2* etc.), or (b) using the fragment descriptors alone instead of the current set of molecular descriptors, or (c) using a subset of the 102 descriptors obtained via LASSO (L1)-based sparse feature selection technique (implemented using the *SelectFromModel* module of sci-kit learn with default parameters), made the model performance significantly worse (with MAE > 0.6 *logK*_1_ units with AdaBoost) thereby establishing that the original 102 descriptors were optimal in mapping the features to the target property. In fact, upon using the LASSO (L1) sparse feature selection technique, the retained subset of descriptors neither included the most important features (see the next sub-section for feature importance analysis) nor the metal and medium related properties. Details of the LASSO (L1) feature selection are provided in the supporting information. The other ML algorithms exhibited comparable performances with test MAE in the range of 0.50 to 0.65 *logK*_1_ units. The only exceptions were SVM (linear) and KRR (linear) having average test MAE of 1.80 and 1.82 *logK*_1_ units, respectively. This highlights the limitation of linear regressors in modelling dependencies between the covariates and response variables on such complex datasets. In fact, an earlier study on protein–ligand binding affinity also exhibited that predictions based on RF and decision trees consistently outperformed linear regression models^35^.

**Table 1:** Evaluation of the ten ML models employed in this work.

Model used	Test R^2^	Test RMSE	Test MAE	Optimized parameters	Normalization
Random forest	0.97	0.94	0.44	*n_estimators* = 60*, max_depth* = 40, *min_samples_leaf* = 2	Normal quantile
KNN	0.95	1.31	0.62	*n_neighbours* = 2, *p* = 2	Robust
SVM (linear)	0.80	2.64	1.80	*C* = 48	Minmax
SVR (RBF)	0.95	1.25	0.57	*C* = 450, γ = 0.073	Uniform quantile
KRR (linear)	0.82	2.51	1.82	*α* = 0.25	Robust
KRR (polynomial)	0.96	1.17	0.60	*α* = 0.030, γ = 0.082	Uniform quantile
KRR (RBF)	0.96	1.17	0.53	*α* = 0.002, γ = 0.006	Robust
KRR (Laplacian)	0.98	0.86	0.43	*α* = 0.001, γ = 0.012	Uniform quantile
MLP	0.96	1.15	0.62	*n*_1_ = 700, *n*_*2*_ = 800	Normal quantile
AdaBoost	0.98	0.91	0.39	*n_estimators* = 20*, max_depth* = 40	Normal quantile

In order to further augment the predictive power of our models, we implemented an automated feature engineering and selection using the *autofeat* library of python^36^. It is a framework inspired by the SISSO algorithm^37^ that automatically generates a large number of non-linear features from the input descriptors and then selects the most informative of them as additional features. The non-linear features are generated in an iterative fashion using algebraic combinations of features with different operators (e.g. + , − ) and filtered using the *FeatureSelector* class of *autofeat* by imposing a significance threshold of univariate feature score. Here, we employed the *autofeat* package on our dataset normalized using the NormalQuantile method, as it gave the best results with AdaBoost. A 2-step feature engineering was performed to generate a total of 255,255 non-linear features, followed by 1-step feature selection that culminated in 330 final features getting selected (including 102 original features), after correlation and noise filtering. However, when the transformed input feature space was fed into AdaBoost model, barely any improvement was observed in the test MAE (0.38 versus 0.39 for untransformed features). Same trend was observed in the case of RF and KRR models. The *R*^2^ for linear regression, however, increased considerably from 0.80 to 0.96. This is rather expected considering that even on benchmark datasets, the model showed remarkable improvements on linear regression tasks but could not outperform the state-of-the-art RF regression models^36^.

Furthermore, a Principal component Analysis (PCA) was carried out which revealed that 18 and 34 principal components captured 95 and 99% of the variance in data, respectively. However, the computed error metrics were significantly worse with all 34 principal components for both decision tree and kernel-based approaches. Therefore, going ahead, we included all 102 original features in our ML computations. Details of the descriptors used were provided in the Methods section.

### Feature importance analysis

**Figure 2:**
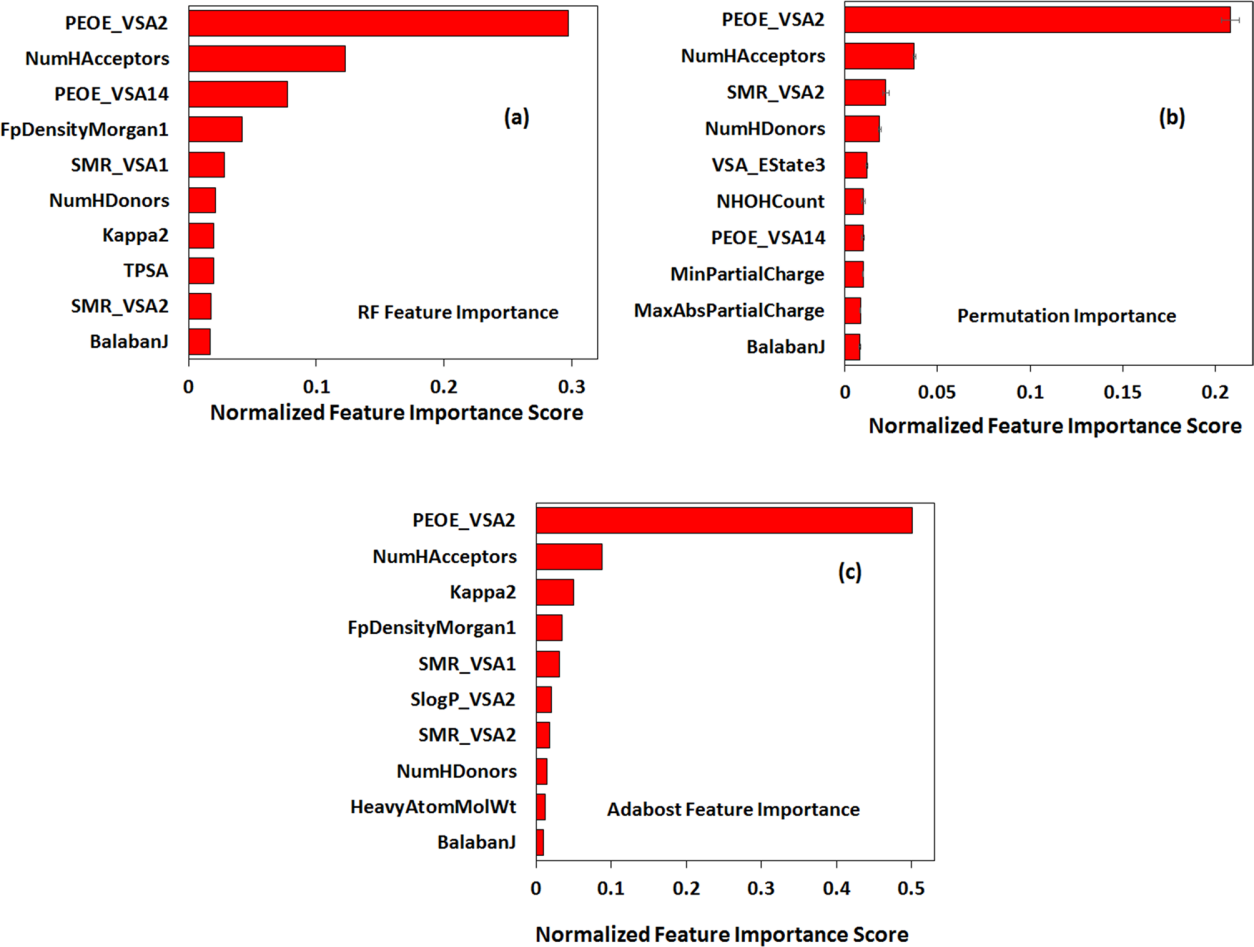
Top 10 highest ranked descriptors based on a variety of feature importance methods: (**a**) random forest; (**b**) permutation importance; and (**c**) AdaBoost.

A feature importance analysis was carried out to obtain the feature rankings of 102 features that were used to train the models. Figure 2a–c show the feature importance scores of ten highest-ranked descriptors using a variety of approaches, namely, Random Forest, Permutation Importance and AdaBoost. Normally, the ensemble methods and decision trees (e.g. Random Forest, AdaBoost) are faster and easier to implement compared to other approaches like LIME^38^. These approaches weigh each feature according to the corresponding mean decrease in impurity, which for regression tasks is the variance. Permutation Importance method provides a different measure of feature importance by incorporating random shuffling and eliminating bias towards high cardinality features in tree-based models. Being model agnostic, it provides a more reliable estimate of the feature rankings.

From the plots Fig. 2a–c, it follows that the RF, Permutation Importance and AdaBoost feature rankings are largely similar with *PEOE_VSA2* and *NumHAcceptors* as the two highest-ranked features. The descriptor *BalabanJ* appears in Fig. 2a–c while descriptors like *Kappa2*, *FpDensityMorgan1*, *SMR_VSA1* etc. are common to Fig. 2a,c. These are 2-D topological/topochemical properties which provide useful information about the molecular surface and its potential interactions with the binding species. For instance, *PEOE_VSA* and *SMR_VSA* capture the atomic contributions based on partial total charge (*PEOE*) and molar refractivity (*SMR*) to the Van der Waals surface area (*VSA*)^39^. Similarly, *Balaban’s J* and *Kappa* are topological indices that come from chemical graph theory^40,41^ while *FpDensityMorgan* generates the similarity fingerprints using certain chemical and connectivity attributes of atoms^42^. Besides molecular descriptors, two other properties—temperature and ionic concentration—ranked among 20 highest-ranked features (not shown in the plots) underlining the importance of experimental conditions in predicting the metal–ligand binding affinity.

### Data distribution and performance on test dataset

**Figure 3:**
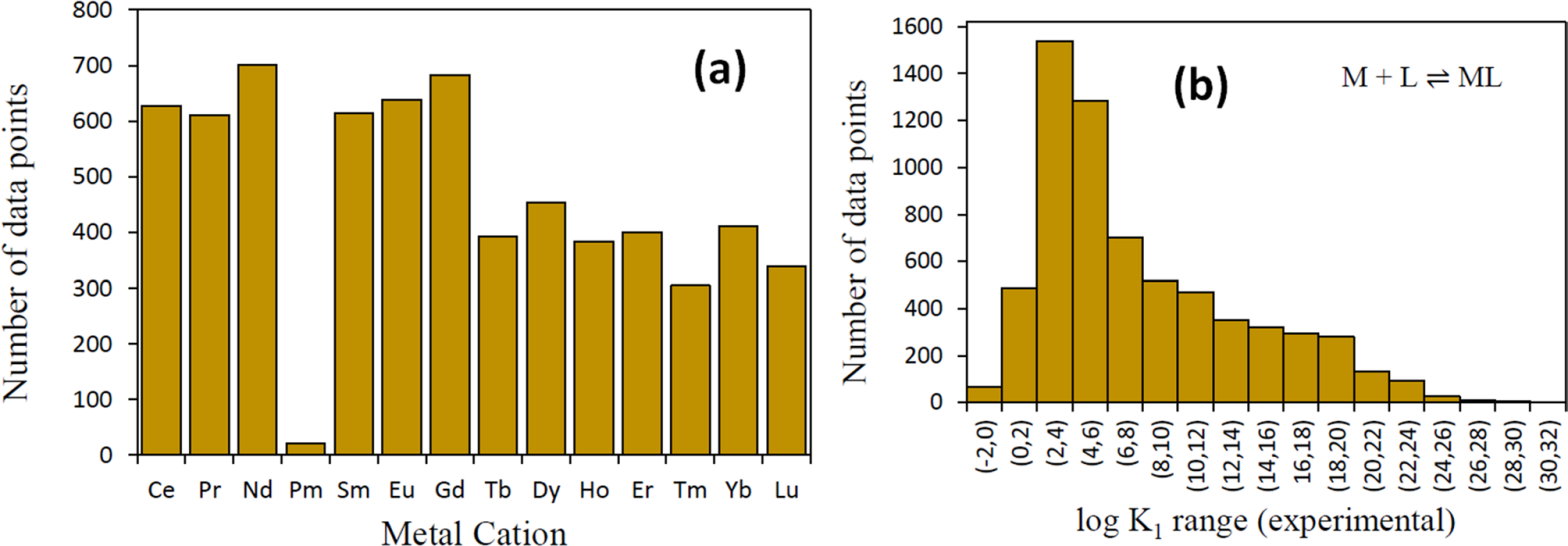
The distribution of data points in the initial lanthanides dataset based on: (**a**) the metal cation and (**b**) the range of *logK*_1_ values.

The distribution of the number of data points per lanthanide cation and experimental values of *logK*_1_ for complexation of metal ions with diverse ligands have been plotted in Fig. 3a,b. Except for Pm containing only 21 entries, all metal cations have more than 300 entries in the dataset. The *logK*_1_ values for all M-L complexes (Fig. 3b) lie in the range of − 1.4 to 30.7 with maximum number of data points lying between 2 and 4 *logK*_1_ units. Almost 70% of the values lie between 0 and 10 *logK*_1_ units, while around 25% and 0.04% of values are in the range of 10 to 20 and 20 to 30 *logK*_1_ units, respectively.

**Figure 4:**
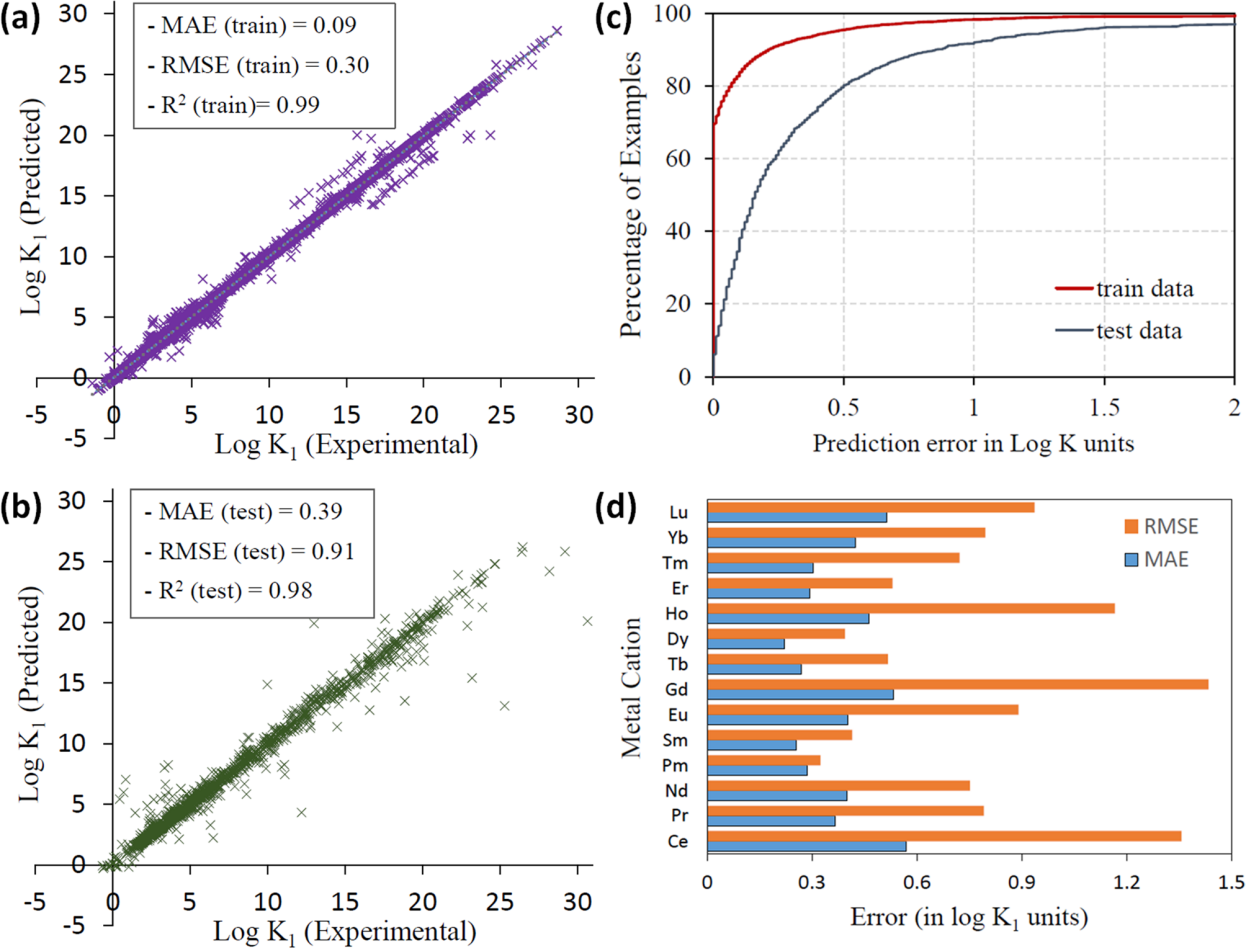
Model predictions on the training and test dataset: (**a**,**b**) show the parity plot between the predicted and experimental *logK*_1_ values, (**c**) shows the regression error curve and (**d**) shows the MAE and RMSE values for individual cations.

Figure 4a,b show the AdaBoost predictions on the train and test datasets as a parity plot between the experimental and predicted *logK*_1_ values. It can be inferred that a small number of large margin outliers fall in the spectrum of high *logK*_1_ values, which is expected considering the skewness in data (Fig. 3b), with only 269 values above 20 *logK*_1_ units. The predictions are further quantified in Fig. 4c, where the percentage of examples has been plotted against the absolute prediction error. Clearly, more than 95% of the test (train) examples have prediction errors of less than 1.5 (0.5) log *K* units, demonstrating excellent predictability of our model. The test MAE (RMSE) for individual metal cations shown in Fig. 4d varies in the 0.2–0.6 (0.3 to 1.4) *logK*_1_ units range, implying that the variation is not too large.

**Figure 5:**
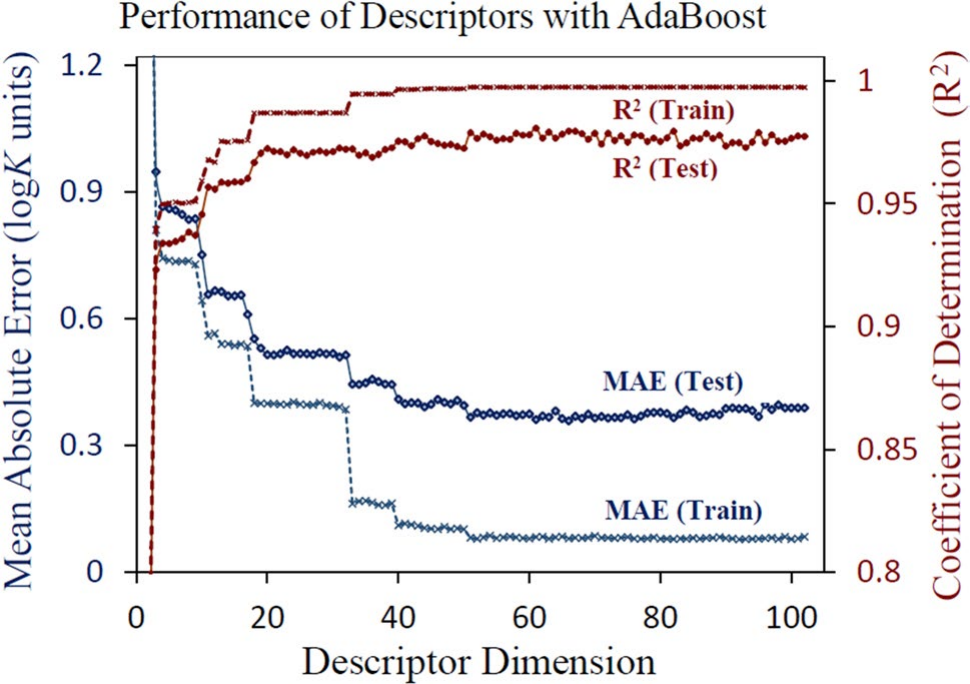
Computed error metrices for the train and test dataset as a function of the dimensionality of the descriptor space.

Figure 5 depicts the computed performance metrics using AdaBoost for the train and the test data set, with increasing number of descriptor dimensions. The features were arranged in the descending order of AdaBoost importance scores and the AdaBoost model with best parameters (listed in Table 1) was employed to evaluate MAE against the dimensionality of feature space. As can be seen, the test MAE reduced to 0.4 *logK*_1_ units while the train MAE dropped down to less than 0.1 *logK*_1_ units, with just 50 top-ranked descriptors. Interestingly, steep reductions in MAE are observed after physically relevant medium and metal properties get included in the model, like ionic concentration (feature 17) and the number of ‘*f* ‘ electrons in metal (feature 32). Beyond 50 descriptors, no significant improvements were observed in the train/test MAE. However, we retained all physically meaningful descriptors like the metal ionization energies, molecular charge and a few topological molecular descriptors, in the interest of better model generalizability and robustness.

**Figure 6:**
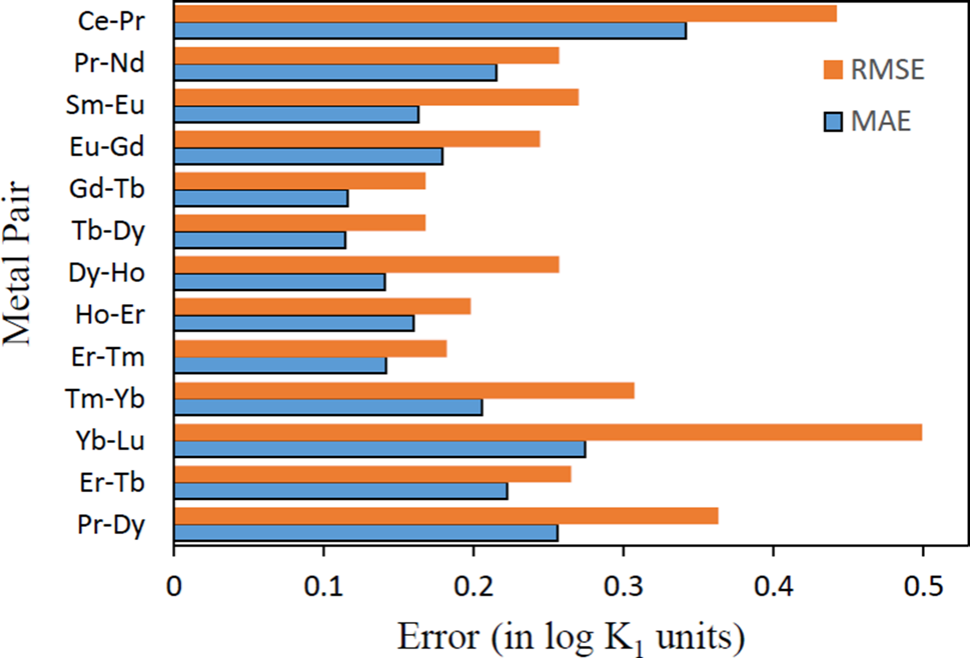
Computed MAE and RMSE in the selectivities of several adjacent lanthanide metal ion pairs.

The MAE and RMSE values of the predicted selectivities of ligands for adjacent pairs of lanthanide cations have been shown in Fig. 6. For any given pair of cations A and B, the higher the difference between *logK*_1_ values for a ligand, the more preferentially it binds to the metal with higher *logK*_1_. Very few ligands have available selectivity data for adjacent pairs of metal cations with the same experimental conditions, i.e. temperature, concentration and solvent medium. Also, the Pr–Dy and Er–Tb pairs were considered despite the cations not lying adjacent to each other, owing to their relevance vis-à-vis recovery of rare earth metals from or e-wastes^43^. Since no common experimental conditions were available for Nd and Dy, the Nd–Dy pair was excluded. Clearly, the predicted values of selectivity show a good match with experimental data, as is evident from the range of MAE (RMSE) values (Fig. 6)—0.11 (0.17) *logK*_1_ units for Tb–Dy and 0.34 (0.44) *logK*_1_ units for Ce–Pr. This is encouraging considering that all MAE (RMSE) values are less than their respective test set values of 0.39 (0.91) *logK*_1_ units. Besides, the models were trained on individual values of *logK*_1_ and not on selectivities, which requires that common experimental conditions be present for a ligand binding with any pair of metal cations.

For testing the generalizability of our model, out-of-sample validation was also performed on six well-known nitrogen donor ligands, the results of which are presented in Table 2. A predicted MAE of 0.95 *logK*_1_ units on this data set points to the good generalizability of our model.

**Table 2:** Comparison of experimental versus predicted *logK*_1_ values for nitrogen donor ligands.

Ligand	Cation	*logK*_1_ (predicted)	*logK*_1_ (experimental)
ADPTZ^45^	Ce^3+^	4.82	4.28
	Pr^3+^	4.76	4.43
	Nd^3+^	4.82	4.62
	Sm^3+^	4.82	4.62
	Eu^3+^	4.69	4.51
	Gd^3+^	4.69	4.29
	Tb^3+^	4.76	4.15
	Dy^3+^	4.69	4.07
	Ho^3+^	4.69	4.05
	Er^3+^	4.69	4.1
	Tm^3+^	4.62	4.23
	Yb^3+^	4.69	4.3
	Lu^3+^	4.74	4.4
MePhPTA^46^	Eu^3+^	6.7	6.95
Phen^46^	Eu^3+^	4.84	4.23
TERPY	Gd^3+^^47^	3.85	2.6
	Lu^3+^^48^	3.5	2.8
	Eu^3+^^48^	4.15	2.4
Me-BTP^49^	Nd^3+^	3.46	2.9
	Eu^3+^	3.81	2.9
PDAM^50^	Ce^3+^	5.94	4.06
	Pr^3+^	5.93	4.09
	Nd^3+^	6.3	4.09
	Sm^3+^	6.32	4.27
	Eu^3+^	6.32	4.17
	Gd^3+^	6.28	4.3
	Tb^3+^	6.26	3.93
	Dy^3+^	6.15	4.05
	Ho^3+^	4.69	3.89
	Er^3+^	4.65	3.84
	Tm^3+^	3.76	3.88
	Yb^3+^	4.66	4.08
	Lu^3+^	4.74	3.8

Carbon, Nitrogen, Hydrogen and Oxygen atoms are shown in cyan, blue, white and red colors, respectively. The molecular images were generated using the VMD 1.9.3 (https://www.ks.uiuc.edu/Research/vmd/vmd-1.9.3) software^44^*.*

### Predictions on PubChem dataset

Having established the predictive power of our framework, we utilized the optimized AdaBoost model for predicting binding affinities of each lanthanide cation with the compounds in the PubChem database^51^. PubChem is a public repository containing the structures of a large number of molecules. To start with, the structure of the molecules was downloaded in the SDF file format from the compound_3D PubChem FTP site^52^. Only one conformation per compound was considered. After eliminating duplicate entries and charged molecules, the structure of the remaining compounds (~ 77 million) was optimized with RDKit and their molecular properties were generated using the procedure outlined in Methods section. Next, we eliminated compounds that fell outside of the applicability domain (AD)^53^ of our ML model. The AD represents a chemical space from which the models are derived and is an important tool for reliable application of ML/QSAR models. While a number of approaches exist to build the AD^54^, we chose a bounding box technique owing to its simplicity. To define the AD, the maximum and minimum values of each of the molecular descriptors for the molecules in our training set was first tabulated. Then, a molecule from the PubChem database was considered to lie within the AD if the value of each of its descriptors lie within the range of the tabulated values of the corresponding descriptor. ~ 71 million molecules from the PubChem dataset were found to lie within the AD of our model. Standard experimental conditions, i.e. a temperature of 298 K, an ionic strength of 0.1 M and a perchlorate medium were assumed for the purpose of predicting *logK*_1_. The final dataset was generated by merging the molecular, metallic and medium properties and was normalized using the NormalQuantile approach, which yielded the best test MAE with AdaBoost. The binding affinity predictions on the pubchem data can be obtained from the authors upon request. The distribution of the predicted *logK*_1_ values for each of the 15 cations is uploaded in the supplementary information.

## Discussion

An often-overlooked aspect during the training of ML models is the choice of the normalization method. Of the six normalization methods implemented in this study—MinMax, Standardized, MaxAbs, Robust, NormalQuantile and UniformQuantile—the first three methods are common but are very sensitive to the presence of outliers^27^. The latter three methods, on the other hand, rely on percentile scores or transformation operations to make the data more Gaussian-like. As a result, they are less influenced by a few numbers of very large-marginal outliers. On the current dataset, we clearly see (Table 1) that the quantile normalization method, which transforms the features to follow a uniform/normal distribution, yielded the best results on most of our ML models.

Also, the demonstrated ML framework is a significant advancement over the previous reports on two counts. One, the performance achieved with AdaBoost model (MAE = 0.39 *logK*_1_ units, RMSE = 0.91 *logK*_1_ units, R^2^ = 0.98) is better in comparison to most of the previous related works^7,10–15,55,56^. For instance, Solov’ev et al.^7^ obtained a test RMSE of greater than 1 *logK*_1_ units for M-L complexes of 6 metal cations using the Substructure Molecular Fragment (SMF) descriptors. In a related study^10^, ensemble modeling of the stability constants of 17 lanthanide and transition metal ions (M) with various organic ligands (L) was performed and the best MAE reported on the six largest datasets was greater than 0.6. Secondly, the individual values of MAE for the lanthanide cations are low, in the range of 0.2 to 0.6 *logK*_1_ units (Fig. 4d), implying good generalizability of our framework. In contrast, the earlier reported QSPR models were trained on available *logK*_1_ values for individual metal cations. While this approach may be feasible for a small number of cations, it is impractical to build one model for each metal ion in the periodic table and given experimental conditions. Moreover, we have in this study a large experimental dataset comprising 6,583 *logK*_1_ values, that encompasses diverse sets of ligands, metal ions and experimental conditions, i.e. temperature and ionic strength. Both these quantities influence the *logK*_1_ values, an example being the abrupt reduction in MAE using AdaBoost after inclusion of ionic concentration as a feature (feature 17 in Fig. 5). The above QSPR studies^7,10–15,55,56^, in comparison, were performed at constant values of temperature (298 K) and ionic strength. To that effect, incorporating the experimental conditions that affect the metal–ligand binding boosts the predictive power as well as the reliability of our framework. Furthermore, it is interesting to note that AdaBoost, with decision trees as the base regressor outperformed other linear and kernel-based ML models in this study, while most of the earlier QSPR studies have relied primarily on Multiple Linear Regression^7,10–15^ for binding affinity predictions. The performance improvement with decision trees is expected considering that they were observed to exploit more structural features and the non-linearity in data in a related study^57–59^. More recently, a study on protein–ligand binding affinity further established that the use of RF-Score with RDKit molecular descriptors improves the predictability of ML scoring functions^35^.

Adding to this discussion, the choice of features vis-à-vis selection of relevant molecular descriptors is critical to the performance of ML models. In that context, our predicted feature rankings (Fig. 2a–c) have a meaningful interpretation. The highest-ranked descriptor based on three of the methods is the *PEOE_VSA2* descriptor. This descriptor computes the sum of van der Waals surface areas of atoms whose partial charges lie in the range of − 0.30 to − 0.25. The partial charges are computed using the Partial Equalization of Orbital Electronegativities (PEOE) method^60^. Similarly, *SMR_VSA* and *NumHAcceptors* properties appear in the top ten highest-ranked features. The former sums the van der Waals surface of atoms based on molar refractivity contributions while the latter quantifies the number of available binding sites in molecule for protonation or deprotonation. In essence, these descriptors capture the topological and physical information pertaining to the ligand molecules. On the contrary, the preferred descriptors in most QSPR studies have been the Substructure Molecular Fragments (SMF)^8,23,24^, which only capture molecular topological information by splitting a molecule into fragments and representing atom/bond sequences. Therefore, by combining both topological and physical features, we improve the predictive power and robustness of our models. This observation resonates with the computed MAE values using (a) the current set of descriptors (0.39 *logK*_1_ units) and (b) only the molecular fragment descriptors of RDKit (0.68 *logK*_1_ units).

Finally, we leveraged the performance of AdaBoost model to predict binding affinities of around 71 million ligands in the PubChem database with all lanthanide metals. As a result of the sheer volume and diversity of the chemical structures in the database, we obtained a continuum of predicted *logK*_1_ values. Supplementary Fig. S1 in the supplementary information shows the distribution of predicted *logK*_1_ values for the binding of lanthanide ions with these ligands. For all cations, the maximum number of values lie in the range of 4–6 *logK*_1_ units with hardly any values above 20 *logK*_1_ units. No *logK*_1_ value was predicted to be negative. Furthermore, all adjacent lanthanide-metal pairs have very similar distributions, which is consistent with the experimentally known small differences in their selectivities.

To our knowledge, no previous QSPR studies have carried out *logK*_1_ predictions on such a large scale. On that account, the sheer volume of the generated data makes it a great resource for enabling rapid screening and design of new metal binders, thus overcoming large costs associated with experiments and conventional molecular modelling techniques. It must be emphasized though that while this work addresses an important problem in Cheminformatics, it suffers from a few limitations. Firstly, the models have been trained on a dataset of 14 lanthanide metals (15 cations including Ce^3+^ and Ce^4+^) with various ligands, thus limiting their applicability to other metal cations. Secondly, in solvent extraction, often one or more ligands bind to a metal ion resulting in a neutral extracted complex stoichiometry of M(L)_n_ (n ≥ 1). Neutral ligands such as PDAM or Phen accomplish this by co-extracting counter-ions (for example nitrate ions) from the aqueous phase to maintain charge neutrality of the extracted complex. On the other hand, acidic ligands (such as D2EHPA (bis-2-ethylhexyl phosphoric acid)) can deprotonate to form charge neutral M(L)_3_ type complexes. Thus, in addition to the *logK*_1_ values, successive binding affinities (*logK*_2_, *logK*_3_ etc.) and the acid dissociation constants for acidic ligands are other important factors in screening metal binders. In a future study, we plan to apply deep transfer learning in order to train models for predicting successive binding constants as well as selectivities, on a much larger dataset, comprising all metal cations in the periodic table. Through this, we can further improve the model transferability and guide future efforts in the screening and development of novel metal binders for various applications.

## Supplementary information

Supplementary Dataset

Supplementary Information

## Data Availability

The data used to fit the ML models is provided as an MS-Excel file in the Supplementary Material. The predicted binding affinities of the lanthanide ions with compounds in the pubchem database can be obtained from the authors upon request.
