# Supplementary material for: Applied machine learning for predicting the lanthanide-ligand binding affinities
Source: Sci Rep. 2020 Aug 31;10:14322. doi: 10.1038/s41598-020-71255-9 (PMC7459320; doi:10.1038/s41598-020-71255-9)
Supplement: Supplementary file 2 — Supplementary Information [file 41598_2020_71255_MOESM2_ESM.docx]

**Supplementary Information for:**

**Applied Machine Learning for Predicting the Lanthanide-Ligand Binding Affinities**

Suryanaman Chaube, Sriram Goverapet Srinivasan* and Beena Rai

TCS Research, Tata Research Development and Design Center, 54-B Hadapsar Industrial Estate, Hadapsar, Pune – 411013, Maharashtra, India

*Email: [s.goverapet@tcs.com](mailto:s.goverapet@tcs.com), [sriram.gs.1987@gmail.com](mailto:Sriram.gs.1987@gmail.com)

**Details on preprocessing and model training**

Normalization was carried out on the generated 102 dimensional feature space using six scaling techniques inbuilt in *scikit-learn* – MinMax, Standardized, MaxAbs, Robust, NormalQuantile and UniformQuantile.

In order to train the models, we carried out an 80:20 train-test split followed by a 10-fold cross-validation on the training data. n-fold cross-validation is a resampling procedure that helps detect and prevent data over-fitting. The *GridSearchCV* object of *sklearn* was used for finding optimal model parameters from an identified hyperparametric space, with MAE as the scoring function. MAE was chosen over RMSE as the scoring metric to diminish the influence of a few high prediction errors that could obscure the quality of predictions on the test dataset [1]. All ML computations were carried out on a 2 x 10-core Intel Xeon 4110 (64 GB RAM) CPU Platform. Initially, ML models namely RF and KNN were run and optimized with respect to average MAE in cross-validation set (over 10-folds). These two methods are non-parametric and easy to fit on the training data. Nonparametric ML algorithms do not make strong assumptions about the form of the mapping function and can, therefore, learn a host of functionals from the training data. For RF regressor, three hyperparameters – number of estimators (*n_estimators*), maximum depth (*max_depth*) and the leaf nodes (*min_samples_leaf*) – were optimized by incrementing *n_estimators* and *max_depth* from 1 to 100 each and *min_samples_leaf* from 1 to 10 (in intervals of 1) amounting to a total of 100,000 combinations. Bootstrapping was excluded in this analysis and default values were chosen for other RF hyperparameters. For KNN, the two hyperparameters – *n_neighbours* (number of nearest neighbours) and *p* (power parameter for Minkowski matrix) – were optimized in the 1 to 20 and 1 to 10 ranges, respectively.

Next, we implemented the SVM, KRR and MLP formulations to make our framework more exhaustive. The SVM and KRR methods learn kernel functions through non-linear transformations in feature spaces and differ mainly in terms of the loss function (epsilon insensitive for SVM versus ridge loss for KRR). For this analysis, SVM models with two kernels (linear, RBF) were evaluated for learning the best C (cost parameter) and *gamma* (defining the influence of a single training example) hyperparameters by iterating them in the 1 to 500 and 0.01 to 0.1 ranges, respectively. Similarly, KRR models were implemented with four kernels – linear, polynomial, RBF and Laplacian – to optimize *alpha* and *gamma* in the 0.001 to 0.1 range each. In the case of MLP, 1-layer and 2-layer network topologies were assessed by systematically varying the number of neurons in each layer from 100 to 1000 (in intervals of 50). A ‘relu’ activation function was used in conjunction with ‘Adam’ optimizer and L2 regularization (alpha = 0.0001) technique. A deeper network than that made the data prone to overfitting and was, therefore, not considered.

Finally, we employed the AdaBoost regressor [2], a meta-algorithm that iteratively combines several weak decision trees to generate a stronger correlation between the features and the data. Being an adaptive algorithm, it is less susceptible than simple decision trees to overfitting and effective in reducing the curse of dimensionality. With decision tree as a base regressor, an extensive *GridSearchCV* search was performed by varying *n_estimators* and *max_depth* in the 1 to 100 range each (in intervals of 1), in order to obtain the best AdaBoost model.

(e)

(f)

**Dimensionality reduction via LASSO (L1) sparse feature selection method**

The molecule, metal and medium properties together constituted 102 features to describe the metal ligand binding affinity (*logK_1_*). LASSO (L1) sparse feature selection technique was used for dimensionality reduction and to verify if good ML models could be obtained with a smaller feature space. We utilized the the *SelectFromModel* module of *scikit-learn* to implement the LASSO(L1) sparse feature selection method. The sparsity in the LASSO (L1) method was tuned by varying the regularization parameter ‘alpha’. Decreasing the value of ‘alpha’ reduces the restrictions on the feature coefficients, thus reducing sparsity while larger values of ‘alpha’ promote sparsity by penalizing the coefficients more. Using a default value of alpha = 1 reduced the dimensionality of the feature space to 11, but resulted in a poor AdaBoost MAE of 0.73. Most of the key features appearing in the feature ranking plots of Fig. 2 (see main text), like PEOE_VSA_2, NumHAcceptors, NumHDonors, BalabanJ etc. got eliminated. In addition, no metal or medium related properties were present in the reduced set of 11 features. Reducing alpha to 0.1 resulted in a 35-dimensional feature space with an AdaBoost MAE of 0.43. Yet, many crucial metal and medium properties (based on AdaBoost/RF/Permutation feature importance) like the outer shell electrons/electron affinity/atomic radii of cation, density/molar mass of the solvent medium, etc. were not retained, thereby reducing the interpretability of our model. Finally, for alpha=0.001, the dimensionality of the feature space was reduced to 95, with an MAE (RMSE) value of 0.4 (0.93) which is very comparable to the respective values of 0.39 (0.91) with our original model using 102 features. Since LASSO (L1) did not give a significant reduction in the feature dimensionality to obtain a good model, we retained all the original 102 features in our final models.


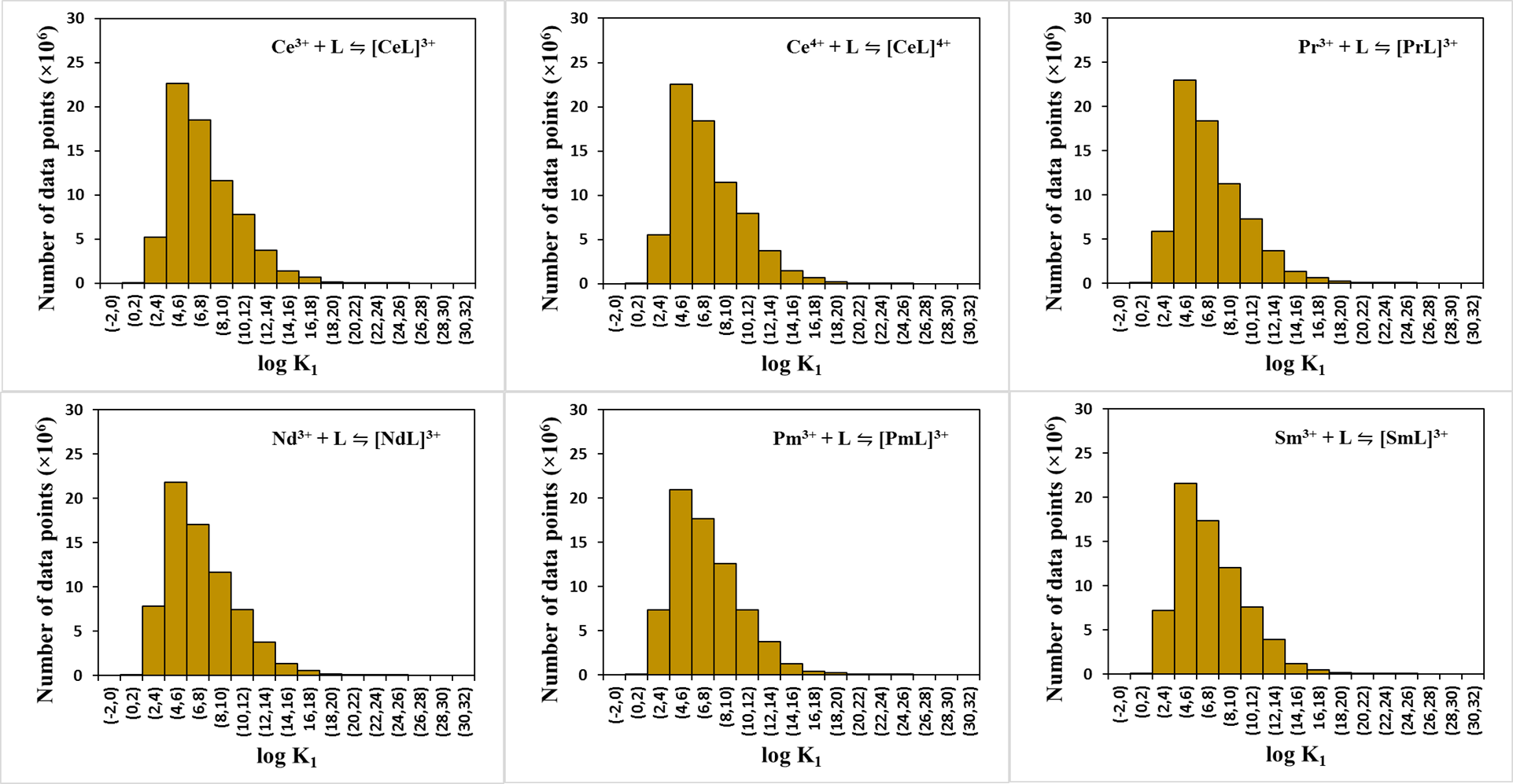


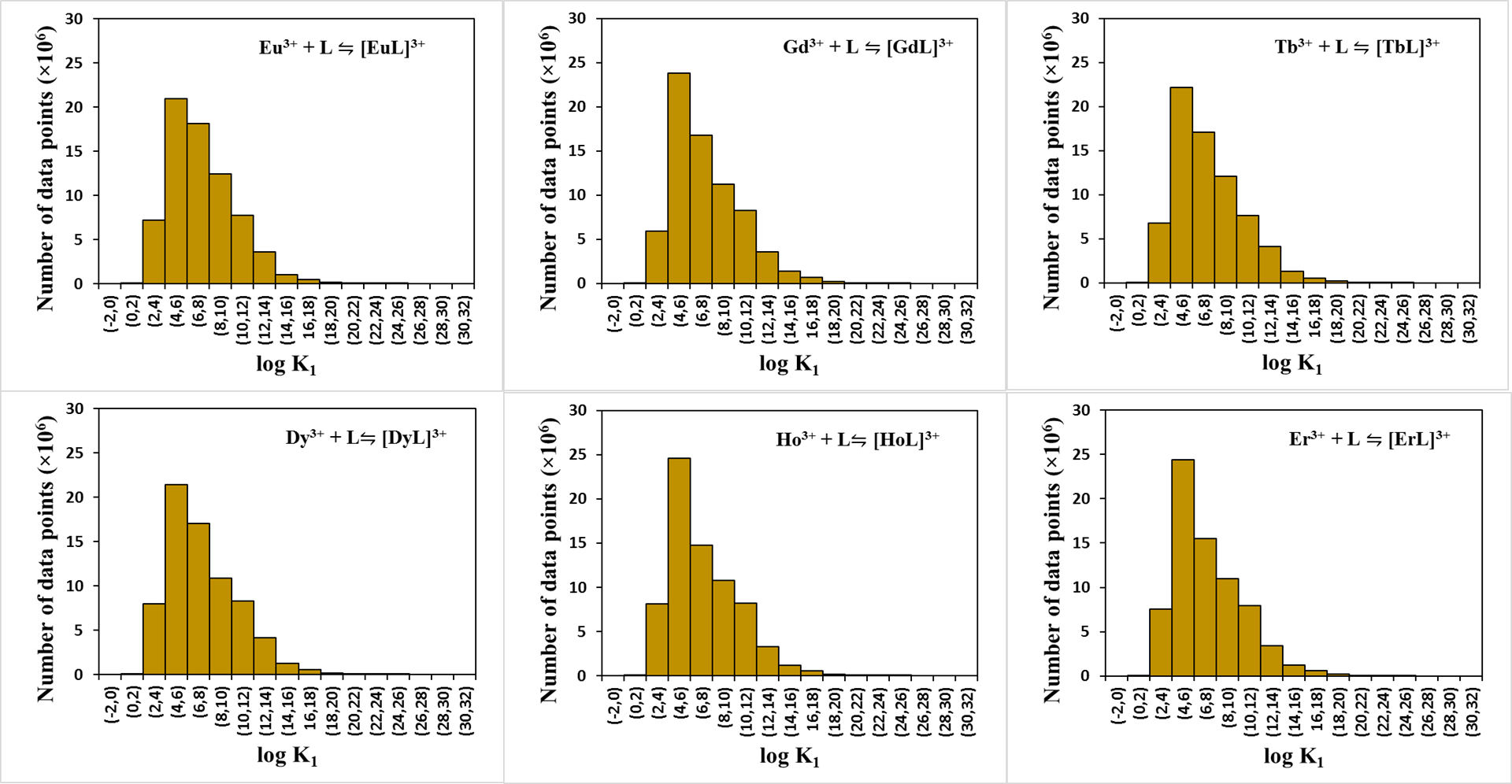


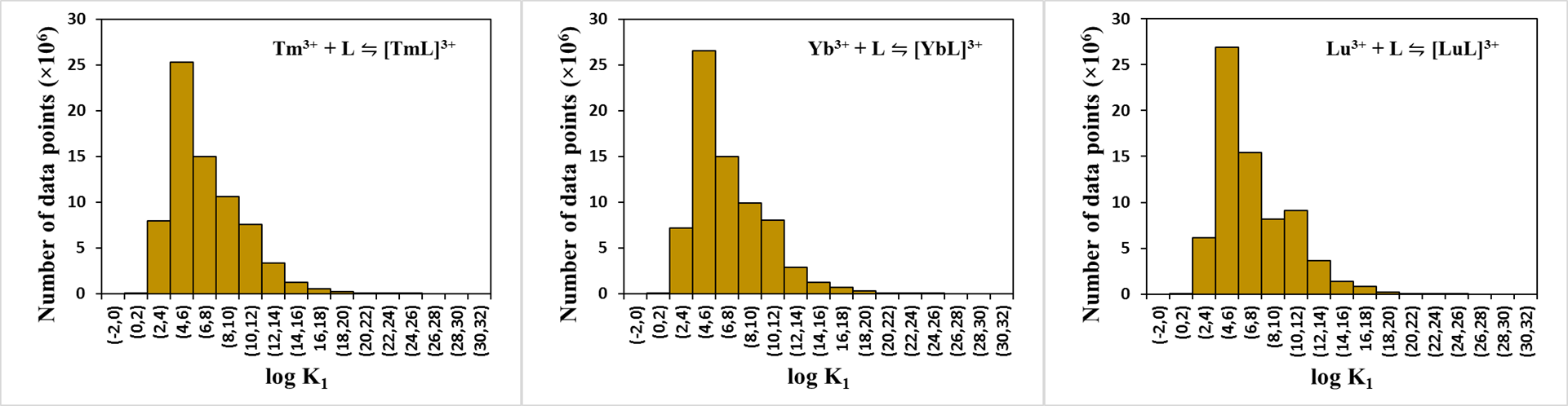


**Supplementary Figures S1:** Distribution of *logK_1_* values for the 15 lanthanide ions binding with all molecules in the PubChem database falling within the Applicability Domain of our best performing AdaBoost model.

**References:**

[1] Roy, K., Das, R.N., Ambure, P. and Aher, R.B., 2016. Be aware of error measures. Further studies on validation of predictive QSAR models. *Chemometrics and Intelligent Laboratory Systems*, 152, pp.18-33.

[2] Freund, Y. and Schapire, R.E., 1995, March. A desicion-theoretic generalization of on-line learning and an application to boosting. In European conference on computational learning theory (pp. 23-37). Springer, Berlin, Heidelberg.
